# Fulminant hepatitis secondary to nivolumab in a patient with Hodgkin’s Lymphoma after complete remission

**DOI:** 10.1016/j.htct.2025.103983

**Published:** 2025-09-25

**Authors:** Ana Júlia Negrelli dos Santos, Arthur Gomes Oliveira Braga, Talita Maira Bueno da Silveira

**Affiliations:** a Faculdade Israelita de Ciências da Saúde Albert Einstein, São Paulo, SP, Brazil; b A.C.Camargo Cancer Center, São Paulo, SP, Brazil; c Irmandade da Santa Casa de Misericórdia de São Paulo, São Paulo, SP, Brazil

Dear Editor,

Hodgkin lymphoma (HL) is a hematologic malignancy with a high cure rate, particularly after first-line treatment based on immunochemotherapy [1]. For relapsed or refractory (R/R) HL, which affects 10–30 % of patients depending on their initial staging, the therapeutic approach often includes second-line regimens consolidated with autologous stem cell transplantation. Immunotherapy, particularly programmed death 1 (PD-1) inhibitors, has emerged as an effective option for achieving long-term disease control in these cases [2].

Nivolumab, a PD-1 inhibitor, is an approved therapy for R/R HL and has demonstrated significant clinical efficacy. However, it is associated with a spectrum of adverse events, including fatigue, rash, loss of appetite, nausea, diarrhea, arthralgia, and elevated transaminases [3,4]. More severe toxicities, such as pneumonitis and autoimmune hepatitis, have also been reported, particularly in solid tumor oncology [5].

There is a current discussion regarding the role of autologous transplantation in patients receiving PD-1 inhibitors (e.g., nivolumab, pembrolizumab). Specifically, the debate centers on the optimal time to discontinue therapy in two distinct patient groups: those who do not undergo consolidation therapy and those who achieve only partial remission after two years. The case reported here serves as a basis to discuss at what point we should start worrying about the extent of long-term treatment with PD-1 and its risks, especially in patients who are in complete remission.

## Case presentation

A 28-year-old man was diagnosed with advanced-stage classical HL with a high International Prognostic Score (IPS >2) in January 2017. This patient received five irregular cycles of first-line eBEACOP-D (escalated bleomycin (Bleomycin), etoposide (Etoposide), Doxorubicin, Cyclophosphamide, Vincristine, Procarbazine, and Prednisone plus dacarbazine) therapy with poor adherence and presented with a primary refractory disease. Subsequent salvage therapies with IGEV (Ifosfamide, Gemcitabine, Vinorelbine, and Prednisolone), DHAP (Dexamethasone, High-dose Ara-C [cytarabine (Cytarabine)], and Platinol [Cisplatin]), and brentuximab showed suboptimal responses due to poor adherence. In 2020, nivolumab was initiated as monotherapy, and, after six cycles, he finally achieved complete remission. Despite irregular follow-up, clinical remission was maintained while continuing monthly nivolumab therapy. Since the patient did not undergo imaging to assess disease response after two years, nivolumab therapy was continued.

In October 2023, three years after achieving complete remission, the patient suddenly started with nausea, vomiting, right upper quadrant abdominal pain, dyspnea, and myalgia, necessitating hospitalization. Laboratory tests revealed fulminant hepatitis with markedly elevated transaminases (AST 3108 U/L, ALT 2380 U/L), canalicular enzymes (alkaline phosphatase 312 U/L, GGT 238 U/L, total bilirubin 20.4 mg/dL), and coagulopathy (INR: 4.49).

The patient underwent a series of laboratory tests that showed no sign of psychoactive substance use, alcohol abuse, use of other medications, or any concomitant infectious condition. The hepatology team described the main hypothesis in this case as an autoimmune fulminant hepatitis secondary to the use of nivolumab. Despite supportive care, he progressed to liver failure, multiorgan dysfunction, and refractory shock, leading to death within days after admission. The patient's response status at the time of death was not assessed.

## Discussion

In 2019, Martins et al. [6] published a review on the adverse effects of the use of checkpoint inhibitors, demonstrating that the frequency of immune-related adverse events related to this kind of medication depends on the agents used, exposure time and the dose. Hepatitis was described as the second most common fatal adverse effect, along with pneumonitis and colitis in patients using PD-1 inhibitors. The review does not describe cases of fulminant autoimmune hepatitis. Nivolumab has also been associated with a well-documented risk of adverse events, including Grade 3 or higher toxicities in approximately 10 % of cases, often necessitating treatment discontinuation [6].

No alternative etiology for the acute liver failure was identified, strongly implicating nivolumab as the causative agent. To our knowledge, this represents the first reported instance of nivolumab-induced fulminant hepatitis in a patient with HL in remission. This case demonstrates that adverse effects from nivolumab can occur, persist, and manifest as severe, life-threatening events even in patients with sustained remission.

## Conclusion

This case underscores the importance of monitoring hepatic function in patients undergoing nivolumab therapy for HL, even those in remission. Early identification of liver dysfunction and prompt intervention are critical to prevent fatal outcomes. High-risk patients receiving immune-checkpoint inhibitors should be regularly monitored by specialized multidisciplinary teams for treatment-related complications, ideally using a personalized surveillance strategy.

This serves as a warning to restart discussions on prolonged therapies with PD-1 inhibitors, the need for ‘chemotherapy holidays’ even for hematologic malignancies and the importance of consolidation as a mark of the end of treatment.

## Conflicts of interest

Authors have no interests that are directly or indirectly related to the work submitted for publication.
